# Comparison of the accuracy of three methods measured the length of the right main stem bronchus by chest computed tomography as a guide to the use of right sided double-lumen tube

**DOI:** 10.1186/s12871-022-01744-z

**Published:** 2022-08-18

**Authors:** Zhuo Liu, Meiqi Liu, Li Zhao, Xiaohang Qi, Yang yu, Shujuan Liang, Xiaochun Yang, Zhongfeng Ma

**Affiliations:** 1 Department of Anesthesiology, First Hospital of Qinhuangdao, N.O. 258, Wenhua Road, Qinhuangdao, Hebei China; 2 Graduate School of Hebei Medical University, Shijiazhuang, Hebei China; 3 Department of Thoracic surgery, First Hospital of Qinhuangdao, N.O. 258, Wenhua Road, Qinhuangdao, Hebei China; 4 Department of General Surgery, First Hospital of Qinhuangdao, N.O. 258, Wenhua Road, Qinhuangdao, Hebei China

**Keywords:** Right sided double-lumen tube, Computed tomography, Carina-proximal distance, Carina-distal distance, Carina-carina distance

## Abstract

**Background:**

The variation of right main stem bronchus leads to the orifice of the right upper lobe bronchus may be obstructed or increase the incidence of malposition intraoperatively when the right sided double-lumen tube is used. Therefore, the aim of this study was to compare the accuracy of three methods measured the length of the right main stem bronchus via chest computed tomography as a guide to the use of right sided double-lumen tube.

**Methods:**

In this study, 168 adult patients undergoing left sided thoracic surgery were included. All these patients were allocated to carina-proximal (C-P) group, carina-distal (C-D) group and carina-carina (C-C) group. The position of endobronchial cuff observed via Fiberoptic bronchoscopy after successful initial placement and after turning the patients to the lateral decubitus position, as well as the incidence of malposition of right sided double-lumen tube intraoperative were recorded to assess the accuracy of three methods in predicting the position of right sided double-lumen tube.

**Results:**

The distance between the carina to the proximal margin of the right upper lobe orifice, carina to the distal margin of the right upper lobe orifice and carina to the first right interlobar carina of the right upper lobe orifice were 17.2 ± 2.3 mm, 25.4 ± 3.7 mm and 28.5 ± 3.1 mm (*P* < 0.05). In the C-D group, the number of endobronchial cuffs seen to be herniating out of the carina, the number of bronchoscopies during initial placement and on the lateral position, the number of total malposition intraoperative and the number of reposition manoeuvres intraoperative were significantly less than the C-P group or the C-C group (*P* < 0.05).

**Conclusions:**

The length of the right main stem bronchus measured by the carina to distal margin of right upper lobe orifice method was more accurate than the other two methods in guiding the use of right sided double-lumen tube.

**Trials registration:**

Clinical Trials. gov. no. NCT04127903. Registered at https://register.clinicaltrials.gov on 16/10/2019.

## Background

Double-Lumen Tube (DLT) is still widely used for One-Lung Ventilation (OLV) [1, 2] and the Right-sided Double-Lumen Tube (RDLT) is mandatory for some specific clinical situations such as distorted anatomy of the left mainstem bronchus for an external or intrabronchial compression, left-sided pneumonectomy, left-sided sleeve resection and left lung transplantation [3], however, the variable anatomy of the right tracheobronchial tree leads to a high risk of right upper lobe bronchial obstruction and collapse [4, 5].

Kim et al. measured the length of right main stem bronchus (RMSB) by “carina-to-distal method”, which they measured the Distance between the tracheal Carina and the Distal margin of the Right upper lobe orifice (DCDR) by chest Computed Tomography scan (CT) as a guide for RDLT use and the results suggested that when the DCDR < 23 mm (the length of the sleeve of RDLT: from the uppermost edge of the endobronchial cuff on the contralateral side of carina to the distal edge of the ventilation slot on the contralateral side of carina) a Left-sided Double-Lumen Tube (LDLT) was more appropriate [6]. However, Kim’s method needed to set two angulations and three lines, which was still a little complex. Bussières JS et al. reported another method measured the length of RMSB, which they called the “carina-to-carina method”. They measured the Distance between the tracheal Carina and the first right interlobar Carina of the Right upper lobe orifice (DCCR) [7]. This method simplified the measurement of RMSB. However, these two methods did not measure the length of RMSB itself, and the accuracy of the two methods had not been compared clinically. So, the aim of this study was to evaluate the accuracy of three methods measured the length of the RMSB by chest CT as a guide to the use of RDLT.

## Methods

This study was approved by the medical ethics committee of the local hospital and written informed consent was obtained from all subjects participating. All methods were performed in accordance with the relevant guidelines and regulations.

**Fig. 1:**
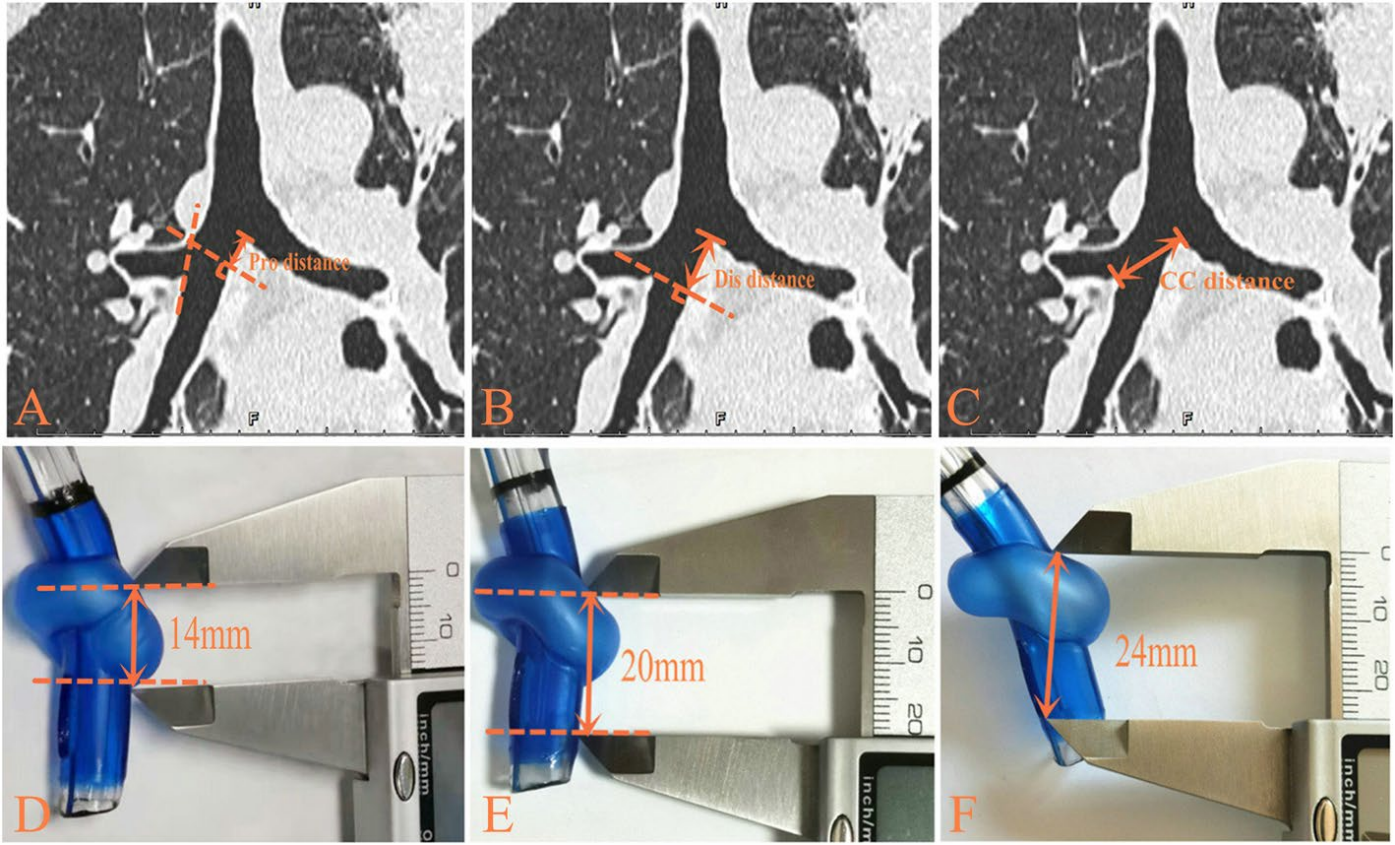
**A** Carina-proximal margin method; **B** Carina-distal margin method; **C** Carina-carina method; **D** The distance between the uppermost edge of the cuff and the proximal margin of the ventilation slot; **E** The distance between the uppermost edge of the cuff and the distal margin of the ventilation slot; **F** The distance between the uppermost edge of the cuff and the distal margin of the ventilation slot on the contralateral side of carina

**Fig. 2:**
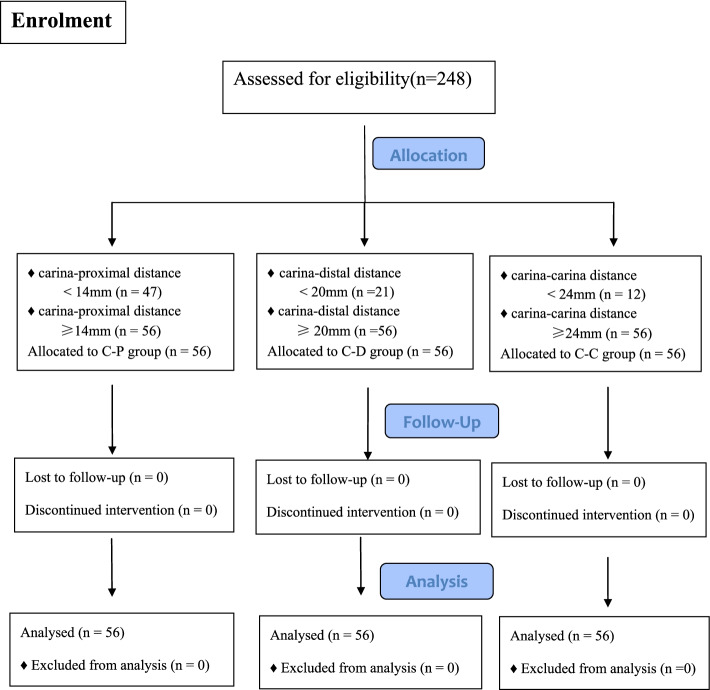
Flowchart

All the patients undergoing elective left sided thoracic surgery and without contraindication to the use of RDLT were included in this study. Exclusion criteria were as follows: age ≤ 18 years or age ≥ 70 years; America Society of Anesthesiologist (ASA) classification > III; distorted anatomy of the tracheobronchial on a chest CT; an intraluminal lesion of the right bronchus; thoracic aortic aneurysm; previous thoracotomy; severe cardiopulmonary disease. The length of RMSB of these eligible patients was measured by an independent anesthesiologist trained by a senior radiologist using Picture Archiving and Communication System (PACS) on a computer according to preoperative chest CT (No CT scan was done specifically for this study). The measurements were made on the coronal plane in the axis of the RMSB, on the frame that a clear carina was showed and the RMSB was on the largest diameter. The enrolled sequence was as follows: First, measured the length of RMSB in eligible patients with Carina-proximal method (measured the distance between the tracheal carina and the proximal margin of the right upper lobe orifice), until there was a patient’s carina-proximal distance ≥14 mm and assigned the patient to Carina-Proximal group (C-P group) (Fig. 1A, D); Second measured the length of RMSB in eligible patients with carina-distal method (measured the distance between the tracheal carina and the distal margin of the right upper lobe orifice), until there was a patient’s carina-distal distance ≥20 mm and assigned the patient to Carina-Distal group (C-D group) (Fig. 1B, E); Third, measured the length of RMSB in eligible patients with carina-carina method (measured the distance between the tracheal carina and the first right interlobar carina of the right upper lobe orifice), until there was a patient’s carina-carina distance ≥24 mm and assigned the patient to Carina-Carina group (C-C group) (Fig. 1C, F). Repeated the above process until the numbers of patients in each group was 56 (Fig. 2).

All the patients were placed in supine position and monitored with Noninvasive blood pressure (NIBP), Heart rate (HR), saturation of pulse oxygen (SpO_2_) and Electrocardiogram (ECG) in the operating room. All the patients were administered with midazolam 0.03mgkg^− 1^, etomidate 0.3mgkg^− 1^, fentanyl 3μgkg^− 1^ and cisatracurium 0.2mgkg^− 1^ for anesthesia induction. All the patients were intubated with a RDLT (Tuoren Medical Technology Company, Xinxiang, China, the size selection was based on the transverse diameter of the narrowest part of the trachea and the right main bronchus measured by chest CT) exactly 3 minutes after intravenous cisatracurium by the same experienced anesthesiologist using a video laryngoscope.

**Fig. 3:**
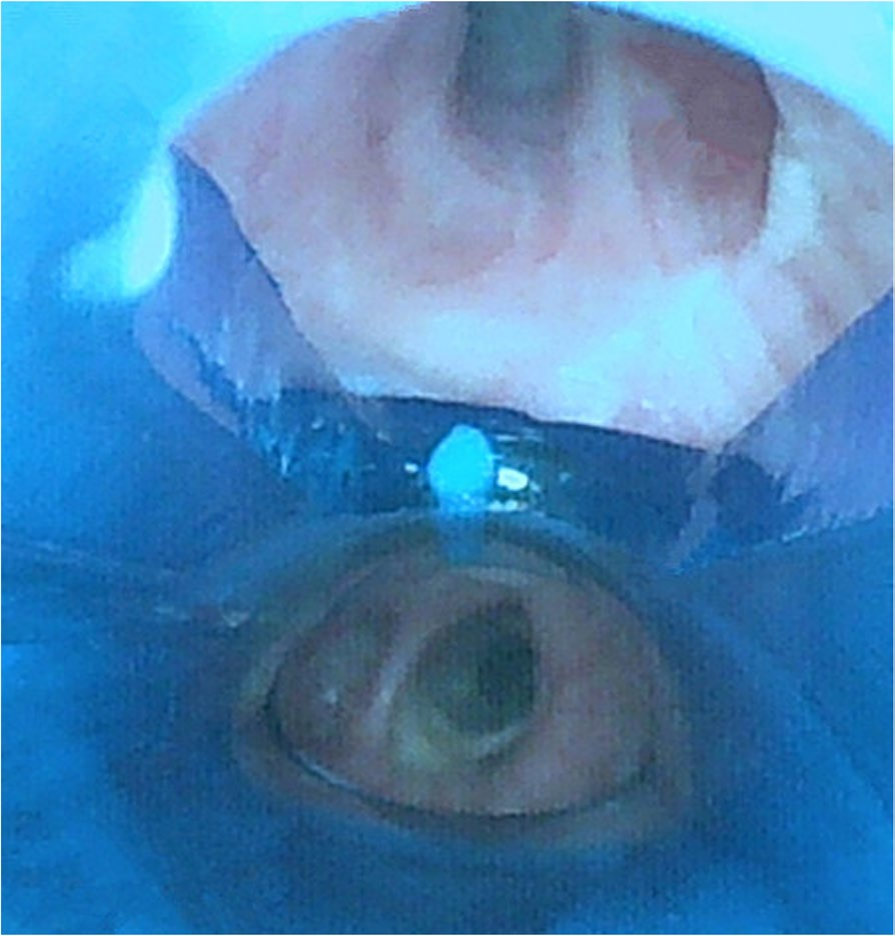
The distal margin of right upper lobe ventilation slot aligned with the distal margin of the right upper lobe orifice

**Fig. 4:**
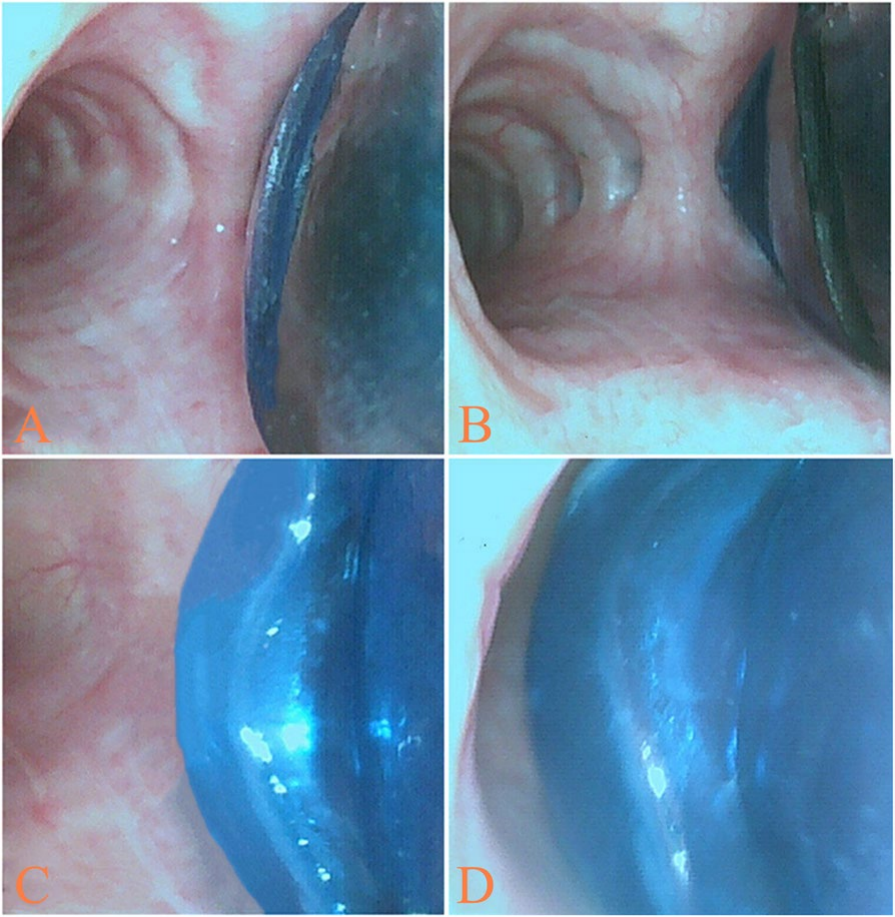
The scores of bronchial cuff position: **A** 1 = invisible; **B** 2 = visible, not herniated; **C** 3 = slight herniation, ventilation maintained; **D** 4 = herniation, inadequate ventilation

The intubation steps were as follows. First, after the tip of RDLT had passed the vocal cords, removed the stylet then advanced the RDLT slightly and rotated 90° toward the RMSB, until a slight resistance was encountered; Second, a Fiberoptic bronchoscope (FOB: external diameter 2.8 mm; MDHAO Medical Technology, Zhuhai, China) was inserted into the right sided lumen of the RDLT to seek for the right upper lobe orifice. If the right upper lobe orifice was not found, retreated the RDLT as sought for the right upper lobe orifice via FOB. If the right upper lobe orifice was not found at the first time, retreated the RDLT into the trachea then inserted the RDLT into the right main bronchus and look for the right upper lobe orifice again during insertion, until the right upper lobe orifice was found and made sure the distal margin of right upper lobe ventilation slot aligned with the distal margin of the right upper lobe orifice (Fig. 3) [7] then inflated the endobronchial cuff (pressure less than 25cmH_2_O). If the right upper lobe orifice was not found after three times, we defined this as intubation failure and a bronchial-blocker tube was used. Third, the FOB was inserted into the left sided tracheal lumen of the RDLT to assess the position of endobronchial cuff at the level of the carina and the scores was recorded (the scores of endobronchial cuff position: 1 = invisible, 2 = visible, not herniated, 3 = slight herniation, ventilation maintained, 4 = herniation, inadequate ventilation) (Fig. 4) [6] then inflated the tracheal cuff (pressure less than 25cmH_2_O).

After deflation of the endobronchial cuff, the patients were turned to the lateral decubitus position. In this process, the RDLT was securely held at the level of the incisors and the patients’ heads were kept in the neutral position. After turning, inflated the endobronchial cuff then checked the positions of the right upper lobe orifice and the positions of endobronchial cuff via FOB again. If there was a sudden increase of peak airway pressure or poor lung isolation intraoperative, the FOB was inserted into the RDLT to detect the positions of the endobronchial cuff and reposition manoeuvre was performed if the endobronchial cuff herniated and affected the ventilation of left lung.

The primary endpoints were the scores of endobronchial cuff position of the RDLT after initial placement and after turning the patients to the lateral decubitus position. The failure of intubations, the number of total malposition of the RDLT intraoperative, the attempts to check the position of RDLT on supine position and on lateral decubitus position were also recorded. The data was collected and analyzed by an independent anesthesiologist.

In this study, the sample size was determined according to a pilot study, with significance set at 0.05 and power set at 80%, the sample size required to detect the differences of the accuracy of RDLT position was 50 patients in each group. Taking into account that the potential risk of patients excluded from the study for unforeseen reasons we recruited 56 patients each group.

## Statistical analysis

The SPSS 21.0 statistical software was used for statistical analysis. Continuous variables were presented as mean ± standard deviation (SD) and the differences among the groups were compared with ANOVA test. The differences of proportions were analyzed using Kruskal-Wallis test. The differences of the incidence were analyzed with Fisher’s exact test. *P* < 0.05 was considered as statistically significant.

## Results

A total of 168 patients were enrolled and there were no differences in demographic characteristics of patients among three groups. The distance between the carina to the proximal margin of the right upper lobe orifice, carina to the distal margin of the right upper lobe orifice and carina to the first right interlobar carina of the right upper lobe orifice were were 17.2 ± 2.3 mm, 25.4 ± 3.7 mm, 28.5 ± 3.1 mm (*P* < 0.05) (Table 1).

**Table 1:** Demographic characteristics of patients

Characteristics of patients	C-P (*n* = 56) CP ≥ 14 mm	C-D(*n* = 56) CD ≥ 20 mm	C-C (*n* = 56) CC ≥ 24 mm	*P*
Age (years)	54.8 ± 12.5	55.6 ± 12.9	53.4 ± 13.4	0.68
Gender (M/F, n)	36/20	40/16	34/22	0.48
ASA (I/II, n)	23/33	28/28	32/24	0.23
Weight (kg)	67.2 ± 10.7	69.6 ± 9.9	66.5 ± 9.6	0.93
Height (cm)	167.4 ± 8.0	167.7 ± 7.9	166.3 ± 7.9	0.59
BMI (kg/m^2^)	23.9 ± 2.8	24.8 ± 2.9	24.2 ± 3.0	0.79
Time of operation (min)	141.7 ± 59.0	151.2 ± 68.0	165.8 ± 71.0	0.16
Time of OLV (min)	112.0 ± 54.1	113.6 ± 59.3	126.9 ± 61.2	0.34
length of RMSB(mm)	17.2 ± 2.3	25.4 ± 3.7	28.5 ± 3.1	0.00

*ASA* American Society of Anesthesiologists, *BMI* Body mass index, *CP* Carina-proximal distance, *CD* Carina to distal distance, *CC* Carina to carina distance, *OLV* One lung ventilation, *RMSB* Right main stem bronchus

In the C-D group, the number of endobronchial cuffs seen to be herniating out of the carina significantly less than that in the C-P group or C-C group both after initial intubation and after turning the patients to the lateral position (*P* < 0.05) (Table 2).

**Table 2:** Position of the endobronchial cuff of RDLT at initial placement and after turning the patients to the lateral decubitus position

Time of evaluation	Score of endobronchial cuff position	C-P(*n* = 56)	C-D(*n* = 56)	C-C(*n* = 56)	*p*
		CP ≥ 14 mm	CD ≥ 20 mm	CC ≥ 24 mm	
Initial placement (n, %)	1	27(48)	35(62)^ab^	19(34)	< 0.01
	2	18(32)	20(36)	23(41)	
	3	10(18)	1(2)	14(25)	
	4	1(2)	0(0)	0(0)	
Lateral position (n, %)	1	21(37.5)	21(37.5)^ab^	9(16)	< 0.01
	2	24(43)	33(59)	30(54)	
	3	7(12.5)	2(3.5)	14(25)	
	4	4(7)	0(0)	3(5)	

The score of endobronchial cuff position (1 = invisible, 2 = visible, not herniated, 3 = slight herniation, ventilation maintained, 4 = herniation, inadequate ventilation); *CP* Carina-proximal distance, *CD* Carina to distal distance, *CC* Carina to carina distance

^a^*P* < 0.05 compared with C-P group; ^b^*P* < 0.05 compared with C-C group

In the C-D group, the number of bronchoscopies during initial placement and on lateral position, the number of total malposition intraoperative and the number of reposition manoeuvres intraoperative were significantly less than the C-P group or the C-C group (*P* < 0.05) (Table 3).

**Table 3:** Comparison of the number of bronchoscopies during initial placement and on lateral position, total malposition intraoperative and reposition manoeuvre intraoperative

Factor		C-P(*n* = 56)	C-D(*n* = 56)	C-C(*n* = 56)	*P*
		CP ≥ 14 mm	CD ≥ 20 mm	CC ≥ 24 mm	
Number of bronchoscopies initial placement (n,%)	1 time	43(77)	52(93)^ab^	44(78)	0.04
	2time	9(16)	4(7)	11(20)	
	≥3time	4(7)	0(0)	1(2)	
Number of bronchoscopies on lateral position (n,%)	0 time	35(63)	45(80)^ab^	33(59)	0.03
	1time	18(32)	11(20)	20(36)	
	≥2time	3(5)	0(0)	3(5)	
Total malposition Intraoperative (n,%)	0 time	36(64)	45(80)^ab^	31(55)	0.01
	1time	15(27)	10(18)	20(36)	
	≥2time	5(9)	1(2)	5(9)	
Reposition manoeuvre Intraoperative (n,%)	0 time	37(66)	50(89)^ab^	40(71)	0.01
	1time	16(29)	6(11)	14(25)	
	≥2time	3(5)	0(0)	2(4)	

*CP* Carina-proximal distance, *CD* Carina to distal distance, *CC* Carina to carina distance. ^a^*P* < 0.05 compared with C-P group; ^b^*P* < 0.05 compared with C-C group

## Discussion

The variable anatomy of the RMSB is the main reason for the difficulties in properly positioning of RDLT, as well as the risk of misalignment between the origin of the right upper lobe bronchus and the lateral orifice of the RDLT, which may lead to an increased risk of hypoxia, hypercapnia, and atelectasis of the right upper lobe during one-lung ventilation [8, 9]. In order to address this issue, we compared three methods measured the lengths of RMSB to guide the use of RDLT and the results showed that the carina-to-distal margin method was more accurate than the other two methods in guiding the use of RDLT. The reasons may be as follow: First, carina-to-proximal margin method was difficult for the operator to align the proximal margin of right upper lobe ventilation slot of RDLT with the proximal margin of the right upper lobe orifice using FOB for the forward viewing field of FOB was 100°-110° and the space in the endobronchial tube of RDLT was too narrow to adjust the tip of the FOB freely, so in this group we still made sure the distal margin of right upper lobe ventilation slot aligned with the distal margin of the right upper lobe orifice. Therefore, although carina-to-proximal margin method measured the length of the RMSB itself, this method was unable to predict the position of endobronchial cuff accurately; Second, carina-to-carina method simplified the measurement, however, the length of RMSB measured by this method did not exactly represent the actual length of RMSB for this length was closely related not only to the DCDR but also to the diameter of the RMSB, if the inner diameter of the RMSB was significantly different from the outer diameter of the sleeve of RDLT, errors might occur in predicting the position of the endobronchial cuff; Third, carina-to-distal margin method was easy to make the distal margin of right upper lobe ventilation slot aligned with the distal margin of the right upper lobe orifice under the direct vision of FOB, so compared the length of the sleeve of RDLT measured the distance between the uppermost margin of endobronchial cuff on carina side and the distal margin of right upper lobe ventilation slot on carina side (carina side method) with the DCDR (the length of RMSB measured by carina-to-distal method) was more direct and accurate (Fig. 1). So, the carina-to-distal margin method was more accurate than the other two methods in guiding the use of RDLT.

In earlier study, Jonathan et al. focused on the relationship between the length of RMSB and the length of the sleeve of RDLT and they measured the length of RMSB by FOB in vivo, fresh cadavers and lung casts [10], however, these three methods were complicated and limited by certain conditions. Hagihira et al. measured the length of RMSB using the plain X-ray [11], however, with this approach, only 50% of chest radiographs can clearly identify the bronchial outline [12]. As the CT and PACS progressed, chest CT could identify the bronchial wall more precisely than the plain X-ray [13].

In a recent study, Kim et al. measured the DCDR via chest CT as a guide to the use of RDLT and they also measured the length of the sleeve of the RDLT (from the uppermost margin of the endobronchial cuff on the contralateral side of carina to the distal margin of the ventilation slot on the contralateral side of carina) which was 23 mm. Their results showed that when the DCDR < 23 mm a LDLT was more appropriate. However, the operator evaluated the position of the endobronchial cuff main based on the uppermost edge of endobronchial cuff on the carina side, for the operator inserted a FOB through the left side lumen of the RDLT, first and most easily saw the uppermost edge of the endobronchial cuff on the carina side (Fig. 4), therefore, the distance between the uppermost edge of the endobronchial cuff on carina side and the distal margin of right upper lobe ventilation slot on carina side (carina side method) may be more accurate to predict the position of endobronchial cuff. In this study, we measured the length of the sleeve of RDLT by carina side method and this distance was 20 mm (Fig. 1E). The results showed that the DCDR ≥20 mm was more accurate than the other two methods in guiding the use of RDLT. So, in Kim’ study, DCDR ≥23 mm may be too strict, and some patients with the DCDR between 20 mm and 23 mm might be excluded from using a RDLT. In addition, Kim et al. only compared the accuracy of the endobronchial cuff position and the malposition of tube between the DCDR ≥23 mm group and the DCDR < 23 mm group, while our study compared the accuracy of three measured methods in predicting the position of the endobronchial cuff.

Bussières JS et al. revisited the anatomy of the RMSB and they measured the DCCR (measured the length of RMSB by carina-to-carina method) and the DCCR < 27.9 mm was corresponded with the DCDR < 23 mm. However, Bussières JS et al. did not conduct a clinical study to verify the accuracy of this method. In this study, we compared the accuracy of three methods measured the length of RMSB in predicting the position of the endobronchial cuff and the results showed that Bussières’ method was simple, however, not as accurate as the carina-to-distal margin method in predicting the location of the endobronchial cuff.

There were some limitations with our study. First, the DCDR ≥20 mm used as the threshold to predict RDLT malposition in this study that arises from a single manufacturer, therefore, the results could not be applicable with all brands of RDLT. Second, in this study we could not randomize, so the results may be biased.

## Conclusion

The length of the RMSB measured by the carina to distal margin method ≥20 mm via chest CT was more accurate than the other two methods in guiding the use of RDLT.

## Data Availability

The datasets are available from the corresponding author on reasonable request.

## Abbreviations

DLT: Double-Lumen Tube
RDLT: Right-sided Double-Lumen Tube
RMSB: Right main stem bronchus
FOB: Fiberoptic bronchoscope
DCDR: Distance between the tracheal Carina and the Distal margin of the Right upper lobe orifice
CT: Computed Tomography scan
LDLT: Left-sided Double-Lumen Tube
SPSS: Statistical package for social sciences
PACS: picture archiving and communication system
